# Interactions between Upf1 and the Decapping Factors Edc3 and Pat1 in *Saccharomyces cerevisiae*

**DOI:** 10.1371/journal.pone.0026547

**Published:** 2011-10-31

**Authors:** Kylie D. Swisher, Roy Parker

**Affiliations:** 1 Howard Hughes Medical Institute, University of Arizona, Tucson, Arizona, United States of America; 2 Department of Molecular and Cellular Biology, University of Arizona, Tucson, Arizona, United States of America; Victor Chang Cardiac Research Institute (VCCRI), Australia

## Abstract

In *Saccharomyces cerevisiae*, mRNA transcripts with premature termination codons are targeted for deadenylation independent decapping and 5′ to 3′ decay in a quality control pathway termed nonsense-mediated decay (NMD). Critical factors in NMD include Upf1, Upf2, and Upf3, as well as the decapping enzyme, Dcp2/Dcp1. Loss of Upf2 or Upf3 leads to the accumulation of not only Upf1 and Dcp2 in P-bodies, but also of the decapping-activators Pat1, Dhh1, and Lsm1. An interaction between Upf1 and Dcp2 has been identified, which might recruit Dcp2 to the NMD decapping complex. To determine the nature and significance of the Dcp2-Upf1 interaction, we utilized the yeast two-hybrid assay to assess Upf1 interactions with various mRNA decapping factors. We find that although Dcp2 can interact with Upf1, this interaction is indirect and is largely dependent on the Edc3 protein, which interacts with the N-terminal domain of Upf1 at an overlapping, but not identical, site as Upf2. We also found that Pat1 has an independent two-hybrid interaction with the N-terminus of Upf1. Assessment of both reporter and endogenous NMD transcripts suggest that the decapping stimulators, including Edc3 and Pat1, as well as Edc1 and Edc2, are not essential for NMD under normal conditions. This work defines a larger decapping complex involved in NMD, but indicates that components of that complex are not required for general NMD and might either regulate a subset of NMD transcripts or be essential for proper NMD under different environmental conditions.

## Introduction

An important mRNA quality control pathway in eukaryotic cells is the nonsense-mediated decay (NMD) pathway, whereby transcripts with aberrant termination codons are targeted for degradation. This process prevents the potentially toxic build-up of aberrant peptides that can arise from mRNA transcripts with premature termination codons (PTCs) [1]. NMD targets mRNAs with PTCs that can arise by many mechanisms, including poor transcription fidelity, frameshift mutations, and inefficiently spliced intron-containing mRNAs that are transported to the cytoplasm [reviewed in 2]. In the yeast, *Saccharomyces cerevisiae*, transcripts that contain these PTCs are predominantly targeted by deadenylation independent decapping and 5′ to 3′ degradation [3], [4].

In eukaryotes, including mammalian cells, *Drosophila*, *Caenorhabditis elegans* and yeast, the core NMD machinery is comprised of the factors Upf1, Upf2, and Upf3, which are all essential for NMD to occur in the cytoplasm [5], [6], [ reviewed in 7]. Interaction between Upf1 and Upf2 and between Upf2 and Upf3 has been demonstrated previously by yeast two-hybrid analysis [6], [8], [9] and by co-immunoprecipitation experiments [10], [11], [12]. Recently, crystal structures of mammalian Upf2 bound to Upf1 have demonstrated that hUpf2 directly interacts with the N-terminal cysteine-histidine-rich domain of hUpf1 in a unique bipartite manner [13]. *In vivo* analyses of hUpf2 mutations that disrupt this Upf1-Upf2 interaction surface subsequently inhibit NMD of a reporter transcript Globin 6MS2 [13]. Similarly, crystal structures of mammalian Upf3 bound to Upf2 demonstrate that hUpf3 binds hUpf2 just N-terminal of the hUpf1 binding site [14]. These results provide evidence for the importance of the interactions between the three Upf proteins in promoting and regulating NMD.

Along with the core Upf machinery, the decapping enzyme, Dcp2/Dcp1, is also important for NMD in yeast [3], [15]. Loss of Dcp1 or Dcp2 leads to defects in NMD of both endogenous and reporter transcripts [3], [16]. Additionally, loss of Upf2 or Upf3 leads to the accumulation of both Upf1 and Dcp2 in P-bodies [17]. Here, NMD is impaired by the loss of Upf2 or Upf3, but Dcp2 still appears to be recruited to the site of Upf1 localization. Since a Dcp2-Upf1 interaction has been identified by a high-throughput protein-fragment complementation assay (PCA) in yeast [18], one possibility is that this Dcp2-Upf1 interaction recruits Dcp2 to the mRNA for decapping stimulated by Upf1.

Interestingly, loss of Upf2 and Upf3 also leads to accumulation of other factors, including Xrn1, Dhh1, Pat1, and Lsm1 in P-bodies [17]. Dhh1, Pat1, and Lsm1 are all activators of the decapping enzyme *in vivo* [19], [20], [21]. This suggests that a larger decapping complex consisting of the decapping enzyme and its associated factors may form during NMD. Providing further support for this model, interaction between Upf1 and Dhh1 has been seen by the high-throughput PCA in yeast and between Upf1 and Lsm1 by a high-throughput affinity capture and mass spectroscopy assay [18], [22]. However, the available evidence suggests that both Dhh1 and Pat1 are expendable for proper NMD in yeast, as NMD was not impaired in a *dhh1*Δ *pat1*Δ double deletion strain [23]. This does not rule out that Dhh1 or Pat1 may have a redundant role in NMD with other decapping-associated factors.

In yeast, several additional factors exist that associate with the decapping enzyme and also stimulate or enhance its activity. Among these are the enhancers of decapping factors, Edc1, Edc2, and Edc3. These factors are capable of binding RNA and stimulating the activity of Dcp2/Dcp1 [24]–[27]. Previous evidence suggests that Edc3 alone is not essential for proper NMD in yeast [28]. However, it has not been determined if Edc1, Edc2, or Edc3 individually or in some combination can affect NMD. Therefore, it is possible that these stimulators of decapping also comprise the larger decapping complex that co-localizes with Upf1 in P-bodies and may regulate NMD in a redundant manner with other decapping-associated factors in yeast.

Since both Upf1 and Dcp2/Dcp1 are essential for NMD in yeast, we set out to understand the Upf1 interaction with the decapping enzyme and the broader decapping complex. To do this, we utilized the yeast two-hybrid assay to assess interactions of Upf1 with Dcp2, Pat1, Dhh1 and Edc3. We find that the N-terminal cysteine-histidine-rich domain of Upf1 interacts with Edc3, Pat1, and Dcp2 by two-hybrid, although the Dcp2 interaction with Upf1 is largely mediated by Edc3. Interestingly, Edc3 and Upf2 bind to Upf1 at overlapping, but not identical sites.

To understand the function of these interactions, we assessed NMD of endogenous and reporter transcripts upon loss of these decapping stimulators. We also used Upf1 mutants disrupted for Upf1-Edc3 and/or Upf1-Upf2 interaction to assess the role of the Upf1-Edc3 interaction in NMD. The results obtained suggest that the decapping stimulators are not essential for NMD during normal growth conditions. However, given that both Pat1 and Edc3 associate with Upf1, and that decapping-associated factors are localized to Upf1- and Dcp2-containing foci upon loss of Upf2 or Upf3, it is possible that these decapping stimulators affect a subset of NMD transcripts or have essential roles for NMD under different conditions.

## Results

### Dcp2 interacts with the Upf1 N-terminal domain through Edc3

**Figure 1:**
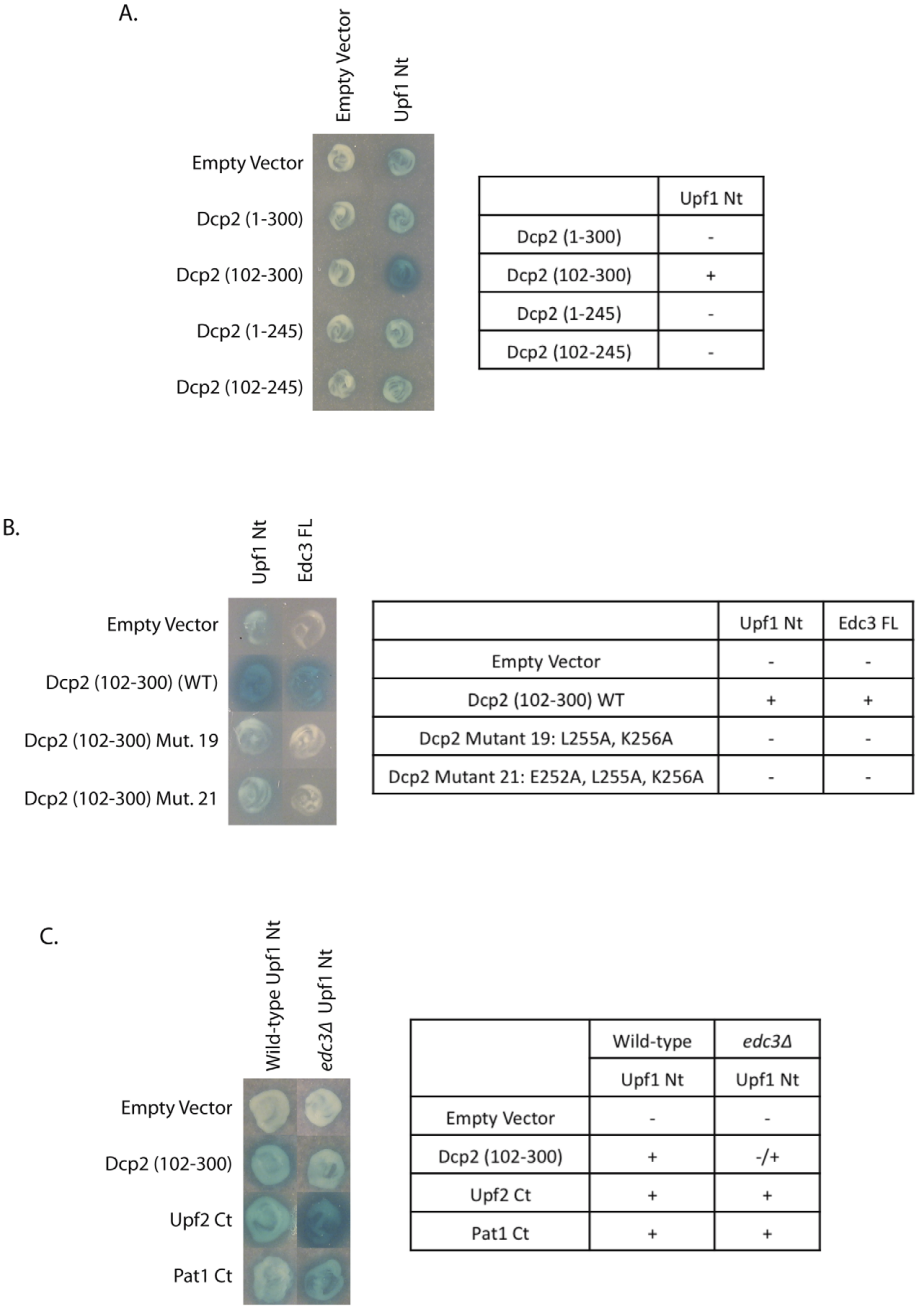
Upf1 and Dcp2 interact in a manner dependent upon Edc3. (A) Upf1 was assessed for its ability to interact by yeast two-hybrid analysis with different domains of Dcp2. (B) The Dcp2-Upf1 interaction was further characterized by yeast two-hybrid analysis at the amino acid level. The structure of *S. cerevisiae* Dcp2 was predicted using the *S. pombe* Dcp2 crystal structure (PDB:2QKM) [31], and point mutations were made along the surface of Dcp2 (102–300) in order to test yeast two-hybrid interaction with Upf1 N-terminus (Nt) and Edc3 full-length (FL). (C) An *edc3*Δ strain was constructed and interaction between the Upf1 Nt and Dcp2 (102–300), Upf2 C-terminus (Ct), and Pat1 C-terminus (Ct) was assessed by yeast two-hybrid analysis.

The first step in understanding how the decapping complex is recruited to Upf1-bound, aberrant PTC-containing mRNAs is to assess if an interaction, whether direct or indirect, exists between Upf1 and the decapping enzyme, Dcp2. While this interaction was found by the high-throughput PCA method [18], we took advantage of the yeast two-hybrid system to assess their association by an alternative method. Yeast Dcp2 is 970 amino acids long but only the first 300 amino acids are required for decapping [16], [29]. Given this, we tested whether we could observe a two-hybrid interaction between Upf1 and either Dcp2 1–300 or Dcp2 102–300. We observed a clear interaction between the Upf1 N-terminal region (binding domain) and the catalytic domain of Dcp2 (residues 102–300) (Figure 1A). We did not see an interaction with Dcp2 1–300, but western analysis showed that this construct was not expressed well (data not shown). No interaction was seen when Upf1 full-length was used, a likely result of either improper folding of the Upf1 protein or blockage of the interaction site when Upf1 full-length was tethered to the β-galactosidase binding domain. For this reason, we utilized the N-terminal cysteine-histidine domain of Upf1, as it is highly conserved and contains the Upf2 binding site [30]. These results identified a two-hybrid interaction between the N-terminal domain of Upf1 and the 102–300 region of Dcp2.

Additional constructs revealed that the 245–300 region of Dcp2, which is an unstructured extension [31], was required for the interaction with Upf1 (Figure 1A). The critical observation was that the 102–300 region of Dcp2 interacted strongly with Upf1, whereas the 102–245 region of Dcp2 did not interact, despite being well expressed based on western analysis (data not shown). Interestingly, this region has previously been identified to contain the amino acids required for interaction with the decapping stimulator, Edc3 [27], [29], raising the possibility that the observed two-hybrid interaction between Dcp2 and Upf1 is through Edc3.

**Figure 2:**
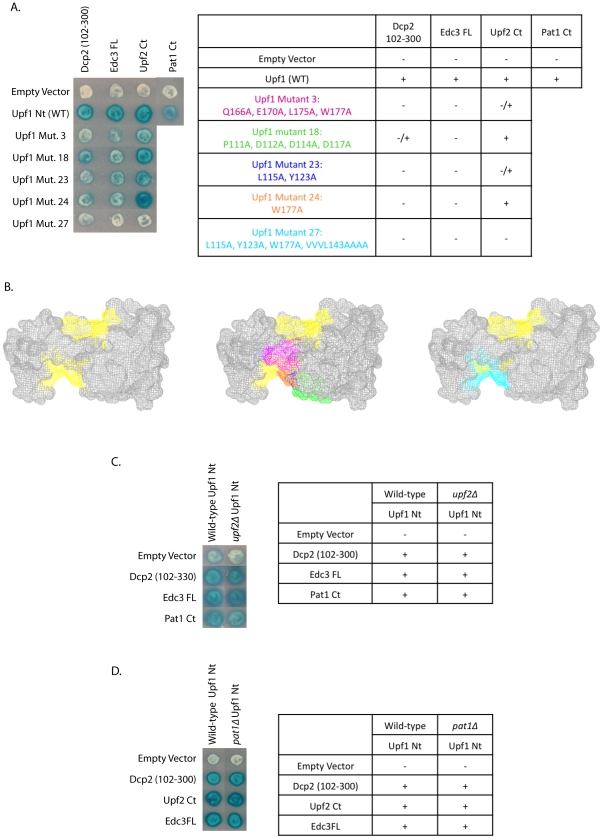
Edc3, Upf2 and Pat1 all interact with the N-terminal domain of Upf1; Edc3 and Upf2 do so in an overlapping, but not identical manner. (A and B) Dcp2 (102–300), Edc3 FL, Upf2 Ct, and Pat1 Ct were all assessed for their ability to interact with Upf1 Nt (WT) by yeast two-hybrid analysis. Interactions between Upf1 Nt and Dcp2 (102–300), Edc3 FL, and Upf2 Ct were further characterized at the amino acid level. The *S. cerevisiae* Upf1 Nt structure was predicted using the human Upf1 crystal structure (PDB:2IYK) [13] and point mutations were made along the surface of Upf1. The predicted Upf2-binding sites are highlighted in yellow. Mutants were tested for interaction with Dcp2 (102–300), Edc3 FL, and Upf2 Ct by yeast two-hybrid analysis. Mutants which showed impaired interaction with Dcp2 (102–300), Edc3 FL, and/or Upf2 Ct are shown in pink, green, dark blue, orange, and light blue colors. (C) A *upf2*Δ strain was constructed and interaction between Upf1 Nt and Dcp2 (102–300), Edc3 FL and Pat1 Ct was assessed by yeast two-hybrid analysis. (D) A *pat1*Δ strain was constructed and interaction between Upf1 Nt and Dcp2 (102–300), Upf2 Ct, and Edc3 FL was assessed by yeast two-hybrid analysis.

Three additional observations confirm that Upf1 interacts with Dcp2 through Edc3. First, Dcp2 mutants L255A K256A (Dcp2 Mutant 19) and E252A, L255A, K256A (Dcp2 mutant 21) are known to disrupt Dcp2-Edc3 interactions [29] and they reduce the two-hybrid interaction of Dcp2 and Upf1 (Figure 1B). Second, the Upf1-Dcp2 two-hybrid interaction is strongly reduced in an *edc3*Δ strain (Figure 1C). This result suggests that Edc3 may function with additional factors to mediate the Upf1-Dcp2 interaction. Finally, we also observed a two-hybrid interaction of Edc3 with Upf1 N-terminal domain (Figure 2A). Taken together, we interpret these results to indicate that Edc3 interacts, directly or indirectly, with Upf1 and thereby can promote a Dcp2-Upf1 interaction.

### The Edc3 binding site of Upf1 overlaps with a predicted Upf2 binding site

To determine how Edc3 interacted with Upf1, we sought to map the interaction site of Edc3 on Upf1 at the amino acid level. To do this, we constructed Upf1 mutants containing one to four alanine substitutions. The crystal structure of mammalian Upf1 (PDB code 2IYK) [13] was used to predict the structure of *S. cerevisiae* Upf1 (Figure 2B), following which the SASA values were calculated using the areaimol program (SASA analysis was kindly provided by John Gross at UCSF). The two Upf2-binding sites were predicted based upon the mammalian Upf1-Upf2 binding data and are highlighted in yellow on the *S. cerevisiae* Upf1 predicted structure (Figure 2B) [13]. The predicted structure of Upf1, along with the SASA values allowed us to construct a series of mutations along the surface of the yeast Upf1 protein between residues 60–208.

Upon testing these Upf1 mutants (binding domain) for interaction with full-length Edc3 (activation domain) by yeast two-hybrid, we identified five mutants that disrupted the Upf1-Edc3 interaction. Mutants 3 (Q166A, E170A, L175A W177A), 18 (P111A, D112A, D114A, D117A), 23 (L115A, Y123A), 24 (W177A), and 27 (L115A, Y123A, W177A, VVVL143AAA) all showed inhibition of the Upf1-Edc3 interaction (Figure 2A). Expression of all five of these mutant proteins was similar or greater than wild-type Upf1, indicating that the defect in interaction was not due to poor expression of the constructs (data not shown). Additional mutants covering the remainder of the Upf1 N-terminal domain surface did not affect the Upf1-Edc3 interaction (data not shown). The defect in Edc3-Upf1 interaction in these mutants defines the regions of Upf1 required for the Edc3-Upf1 interaction.

Interestingly, this putative binding site of Edc3 on Upf1 partially overlaps with the Upf2 binding surfaces. Specifically, mutants 23, 24, and 27 contain alanine substitutions of residues that are predicted to be within one of the two Upf2 binding surfaces (Figure 2; dark blue, orange, and light blue, respectively). Upf1 mutants 3 and 18 reside near this predicted Upf2 binding surface (Figure 2B; pink and green, respectively). These results suggest that Edc3 interacts with Upf1 at or near one of the predicted Upf2 binding sites. We also made amino acid substitutions in the second predicted Upf2 binding surface on Upf1, but were unable to assess their function as they were all poorly expressed (data not shown).

Additionally, we assayed the binding pattern of Dcp2 on Upf1 by the yeast two-hybrid assay. If Edc3 is mediating this interaction, the Dcp2-Upf1 interaction should show a similar binding pattern as Edc3 does on Upf1. Upf1 Mutants 3, 23, 24, and 27 all showed strong inhibition of the Upf1-Dcp2 interaction (Figure 2A). Mutant 18 also inhibited the Upf1-Dcp2 interaction, although to a lesser extent (Figure 2A). Therefore, the mutations that disrupt the Edc3-Upf1 interaction also disrupt the Dcp2-Upf1 interaction, providing further evidence that Edc3 mediates the Upf1-Dcp2 interaction.

### Edc3 and Upf2 share overlapping, but not identical, binding sites on Upf1

Our results suggest that Edc3 interacts with the N-terminus of Upf1 at or near the predicted binding sites of Upf2. To test this, we assayed the binding ability of Upf2 C-terminus with the Upf1 mutants by yeast two-hybrid. As seen in Figure 2A, only Upf1 mutant 27, which contains alanine substitutions for many of the residues in the predicted Upf2 binding surface, showed strong inhibition of the Upf1-Upf2 interaction. Mutants 3 and 23 also showed some weak disruption of the Upf1-Upf2 interaction, while mutants 18 and 24 showed wild-type levels of interaction. These results suggest that the Edc3 and Upf2 binding sites on Upf1 likely overlap, although not perfectly.

To determine if the Upf1-Edc3 interaction was through Upf2, we deleted Upf2 in the yeast two-hybrid strain background and assayed for the ability of Edc3 to interact with Upf1. Loss of Upf2 did not affect the positive interaction between Edc3 and Upf1 (Figure 2C). We also wanted to verify that the Upf1-Upf2 interaction was not affected by Edc3. To test this, we tested the Upf1-Upf2 two-hybrid interaction in an *edc3*Δ strain. Loss of Edc3 did not disrupt the positive interaction of Upf2 and Upf1 (Figure 1C). Interestingly, our yeast two-hybrid results consistently suggested a heightened interaction between Upf1 and Upf2 upon loss of Edc3. These results therefore support the conclusion that Edc3 and Upf2 independently interact at partially overlapping sites on Upf1.

### Upf1 interacts with decay factor Pat1

We have identified an interaction between Upf1 and the decapping-associated factor, Edc3. We therefore wanted to test if Upf1 might interact with other decay factors, such as Pat1 and Dhh1. Pat1 is a translational repressor that can bind to RNA and also stimulate mRNA decapping by Dcp2 [27], [32]. Dhh1 is also a translational repressor that can bind to RNA and activate decapping *in vivo* [21], [27], [33]. We used the yeast two-hybrid system to assay interaction between Upf1 and these two translational repressors.

Our results suggest that Upf1 can interact with Pat1 but not Dhh1 (Figure 2A, data not shown). Specifically, Upf1 interacts with the C-terminal (Ct) domain of Pat1 (Figure 2A). We must note that we were unable to confirm interaction between Upf1 and full-length Pat1, as it showed positive interaction with the empty vector alone. However, the C-terminal domain of Pat1 only showed positive interaction with the N-terminal domain of Upf1, and not the empty vector.

Since both Dcp2 and Upf1 interact with Pat1 at its Ct domain, it is possible that Pat1 also mediates the Upf1-Dcp2 interaction. To test this, we constructed a Pat1 deletion in the yeast two-hybrid strain background and assayed for ability of Dcp2 and Upf1 to interact. Loss of Pat1 did not disrupt interaction of Dcp2 with Upf1 (Figure 2D). Likewise, interaction of Upf1 with both Edc3 and Upf2 was not disrupted by loss of Pat1 (Figure 2D). This suggests that Pat1 does not recruit the decapping enzyme to Upf1, nor does it mediate the Upf1-Edc3 and Upf1-Upf2 interactions.

We also wanted to verify that the Pat1-Upf1 interaction was not through another known Upf1-interacting protein. To determine this, we tested the Pat1-Upf1 interaction in *upf2*Δ and *edc3*Δ strains. Pat1 was still able to associate with Upf1 in the absence of either Upf2 (Figure 2C) or Edc3 (Figure 1C). Therefore, Pat1 is yet another factor that may directly associate with the N-terminal domain of Upf1.

Taken together, we have identified independent two-hybrid interactions of the Upf1 N-terminal domain with Edc3 and Pat1, which are also independent of the Upf2-Upf1 interaction. The Edc3-Upf1 interaction partially overlaps with the Upf2 binding site on Upf1. Finally, the interaction of Edc3 with Upf1 can promote a Dcp2-Upf1 interaction, at least in the two-hybrid assay.

### Upf1-Edc3 and Upf1-Pat1 interactions are not required to promote NMD

The interactions between Edc3, Pat1, and Upf1 are not simply required for NMD since both *edc3*Δ and *pat1*Δ strains show normal NMD [28], [34]. However, it remained possible that Edc3 and Pat1 have redundant roles in recruiting Dcp2 to Upf1 in NMD, which would suggest that an *edc3*Δ *pat1*Δ double mutant might have a defect in NMD.

**Figure 3:**
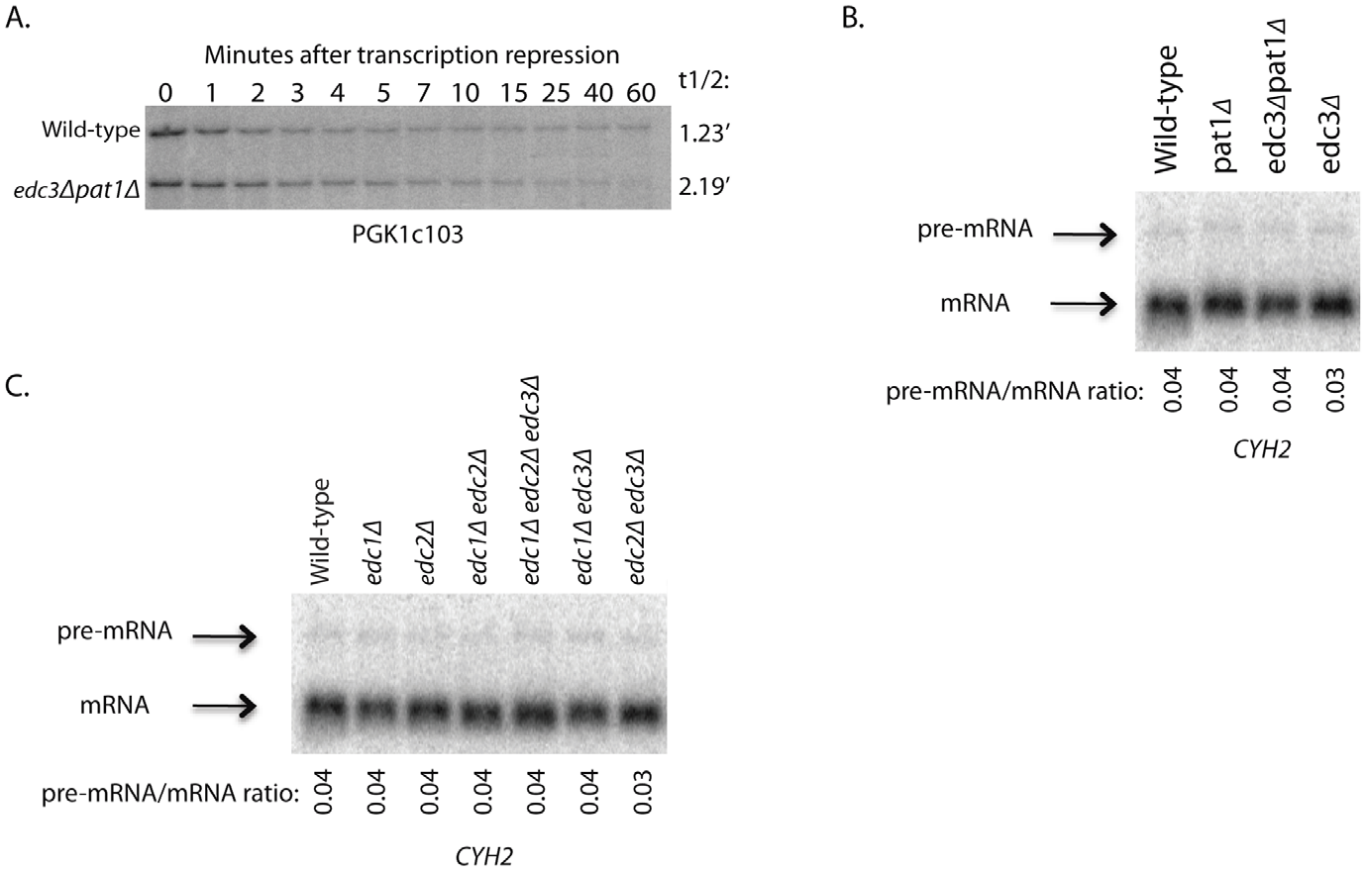
Decapping stimulators, Edc3, Pat1, Edc1, and Edc2 do not promote NMD. (A) NMD of the galactose-inducible reporter transcript containing a PTC, gal-PGK1c103, was assessed in a wild-type and an *edc3*Δ *pat1*Δ double deletion strain over the course of one hour. (B and C) Steady-state pre-mRNA/mRNA ratios of the endogenous NMD transcript, *CYH2*, were assessed in wild-type, *pat1*Δ, *edc3*Δ *pat1*Δ, *edc3*Δ, *edc1*Δ, *edc2*Δ, *edc1*Δ *edc2*Δ, *edc1*Δ *edc2*Δ *edc3*Δ, *edc1*Δ *edc3*Δ, and *edc2*Δ *edc3*Δ strains.

To determine if Edc3 and Pat1 could have a redundant role in NMD, we utilized a galactose-inducible PGK1c103 NMD construct. This construct contains an added sequence that allows for detection of the PTC-containing PGK1c103 independent from the normal PGK1 transcript that is present in the *edc3*Δ *pat1*Δ strain [3]. Our results suggest that Edc3 and Pat1 together do not have a significant role in promoting normal NMD, as degradation of PGK1c103 in the *edc3*Δ *pat1*Δ is only very slightly slower than degradation in the wild-type strain (Figure 3A). Additional analysis of *CYH2* steady-state pre-mRNA/mRNA ratios in *edc3*Δ *pat1*Δ and wild-type strains showed normal levels of NMD upon loss of both Edc3 and Pat1 (Figure 3B).

We also tested if Edc3 might be redundant with Edc1 or Edc2, which can also directly stimulate activity of Dcp2 [24], [25], [27], and observed that even the triple mutant *edc1*Δ *edc2*Δ *edc3*Δ showed normal NMD (Figure 3C). These results, combined with the fact that single deletions of Edc3 and Pat1 have no affect on NMD suggest that the Edc3-Upf1 and Pat1-Upf1 interactions are not required for NMD.

**Figure 4:**
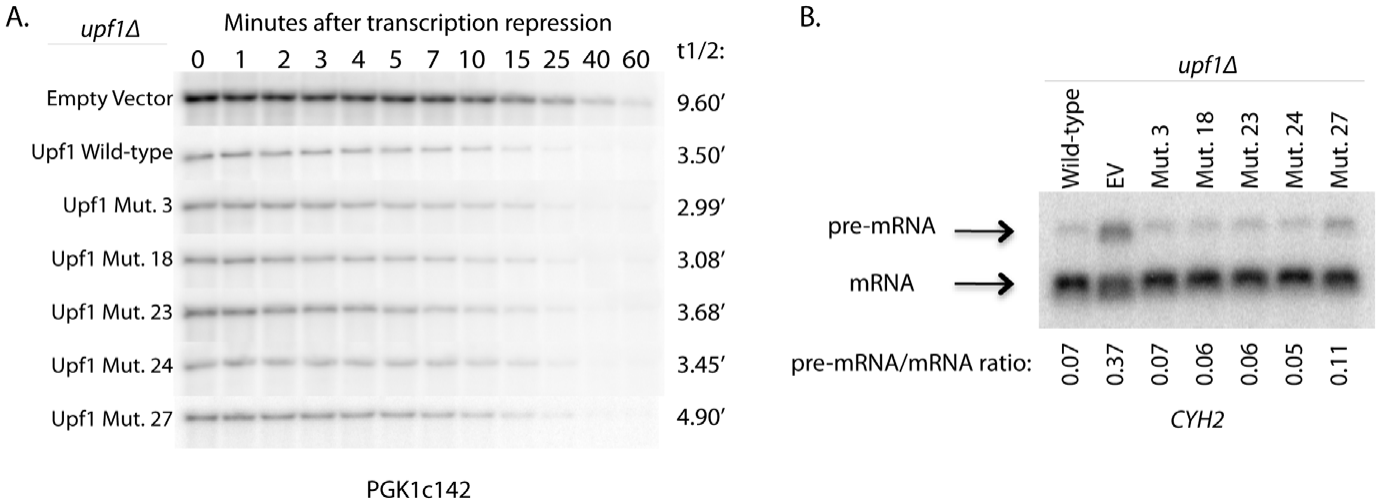
Edc3-Upf1 interaction does not promote NMD. Upf1 mutants that disrupt the Upf1-Edc3 interaction (mutants 3, 18, 23, and 24) or the Upf1-Edc3 and Upf1-Upf2 interaction (mutant 27) were assessed for their role in NMD. (A) Decay of a reporter transcript containing a premature termination codon (PTC) at codon 142 under a galactose-inducible promoter, gal-PGK1c142, was assessed in a *upf1*Δ strain containing either empty vector, Upf1 wild-type, Upf1 mutant 3, Upf1 mutant 18, Upf1 mutant 23, Upf1 mutant 24, or Upf1 mutant 27 on a plasmid. (B) Steady-state pre-mRNA/mRNA ratios of the endogenous NMD transcript, *CYH2*, were assessed in a *upf1*Δ strain containing either Upf1 (WT), empty vector, Upf1 mutant 3, Upf1 mutant 18, Upf1 mutant 23, Upf1 mutant 24, or Upf1 mutant 27 on a plasmid.

Consistent with Edc3-Upf1 interactions not being required for NMD, we also observed that mutations in Upf1 that disrupted interactions with Edc3 did not affect the decay of a PGK1 nonsense-containing reporter, gal-PGK1c142. Specifically, Upf1 mutants 3, 23, and 24, which disrupt the Edc3-Upf1 interaction to a significant extent but have little to no effect on the Upf1-Upf2 interaction, showed no change in decay rate of PGK1c142 compared to wild-type Upf1 (Figure 4A). Mutant 27, which disrupts the Upf1 interaction with both Edc3 and Upf2 by yeast two-hybrid analysis, showed a small defect in the decay rate of PGK1c142 compared to wild-type Upf1 (t1/2 = 3.5 minutes for wild-type and 4.9 minutes for Upf1 mutant 27, Figure 4A). Similar results were also obtained when the levels of the pre-CYH2 mRNA were compared, as Upf1 mutant 27 was the only mutant to show an increase in pre-mRNA levels (Figure 4B). These results suggest that the Upf1-Edc3 interaction is not required for recruitment of Dcp2 to the NMD substrate and confirm earlier studies that suggest a requirement of Upf2 interaction with Upf1 for proper NMD [8], [13].

### Edc3-Upf1 interaction does not negatively regulate NMD

The partial overlap of the Edc3 and Upf2 binding sites on Upf1 suggested the hypothesis that Edc3-Upf1 interaction might be a negative regulatory interaction that inhibits decapping in NMD and this negative regulation would be relieved by Upf2-Upf1 interaction competing with the Edc3-Upf1 interaction. In this model, the purpose of the Edc3-Upf1 interaction is not to simply recruit Dcp2 to an mRNA target, but instead, to inhibit decapping until a specific time designated by the release of Edc3 from its association with Upf1 due to the Upf1-Upf2 interaction. Therefore, we reasoned that loss of the Edc3-Upf1 interaction should suppress any defect in NMD triggered by impaired Upf1-Upf2 interaction. In an attempt to determine if the Edc3-Upf1 interaction is inhibitory and if Upf2 is the ‘switch’ that releases Edc3 from Upf1, we utilized two different assays.

**Figure 5:**
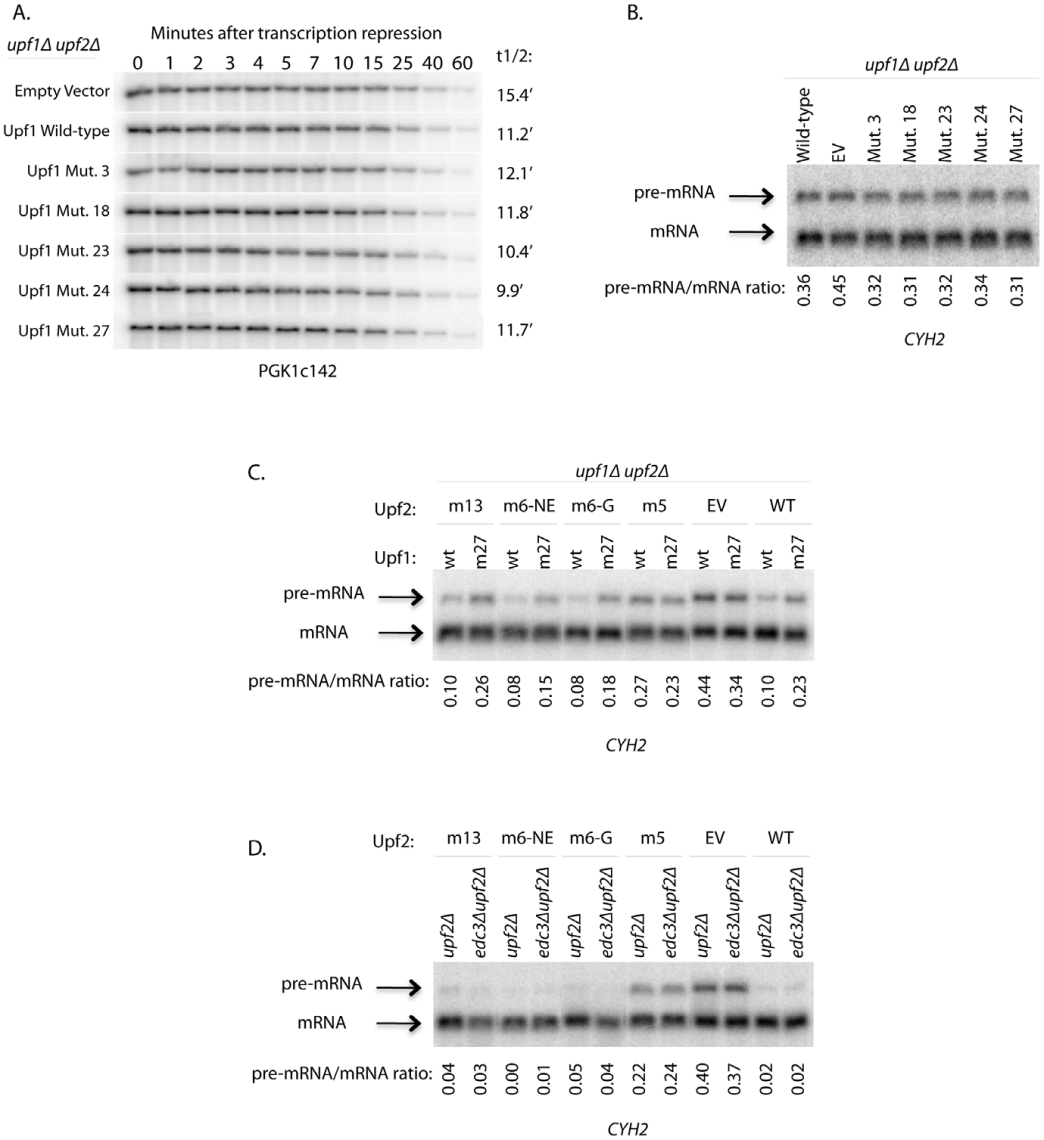
The Edc3-Upf1 interaction is not a negative regulator of NMD. (A) NMD of the PTC-containing galactose-inducible reporter transcript, gal-PGK1c142, was assessed in a *upf1*Δ *upf2*Δ double deletion strain containing either empty vector, Upf1 wild-type, Upf1 mutant 3, Upf1 mutant 18, Upf1 mutant 23, Upf1 mutant 24, or Upf1 mutant 27 on a plasmid. (B) Steady-state pre-mRNA/mRNA ratios of the endogenous NMD transcript, *CYH2*, were assessed in the *upf1*Δ *upf2*Δ strain containing either wild-type Upf1, empty vector, Upf1 mutant 3, Upf1 mutant 18, Upf1 mutant 23, Upf1 mutant 24, or Upf1 mutant 27 on a plasmid. (C) Steady-state pre-mRNA/mRNA ratios of *CYH2* were assessed in a *upf1*Δ *upf2*Δ strain containing either wild-type Upf1 or Upf1 mutant 27 and either Upf2 mutant 13, Upf2 mutant 6-NE, Upf2 mutant 6-G, Upf2 mutant 5, empty vector, or Upf2 wild-type plasmids. (D) Steady-state pre-mRNA/mRNA ratios of *CYH2* were assessed in a *upf2*Δ and an *edc3*Δ *upf2*Δ strain containing either Upf2 mutant 13, Upf2 mutant 6-NE, Upf2 mutant 6-G, Upf2 mutant 5, empty vector, or Upf2 wild-type plasmids.

First, we tested the decay rate of the nonsense codon transcript, PGK1c142, in the presence of Upf1 mutants defective in the interaction with Edc3 and in the absence of Upf2. If decay rates are significantly altered with the Upf1 mutants compared to wild-type Upf1 in a *upf2*Δ strain background, it would suggest that Upf2 functions as the ‘switch’ to trigger Edc3 release. Additionally, the absence of Upf2 allows for easier detection of changes in decay rates due to its impairment of NMD [6], [17]. We monitored decay of PGK1c142 by galactose shut-off experiments to assay the effect of our Upf1 mutants in the *upf1*Δ* upf2*Δ strain. The decay rates of PGK1c142 were comparable between wild-type Upf1 and all five of the Upf1 mutants tested (Figure 5A). Additionally, *CYH2* steady-state pre-mRNA/mRNA levels were comparable between wild-type Upf1 and the Upf1 mutants in the *upf1*Δ *upf2*Δ background (Figure 5B). These results suggest that the Edc3-Upf1 interaction is not inhibitory and relieved by the Upf1-Upf2 interaction.

Second, we analyzed decay of *CYH2* in the presence of Upf2 mutants defective for Upf1 association, NMD, or both. Upf2 may have an additional role in NMD independent of the Upf1-Upf2 interaction, so we utilized Upf2 mutants to better assess the requirement for a ‘switch’ in NMD. Thus, in the presence of the Upf2 mutants defective in NMD it is possible that we can sidestep the requirement for the ‘switch’ by disrupting the Edc3-Upf1 interaction. This should potentially lead to the suppression of the NMD defects caused by mutations in Upf2. To do this experiment, we compared the steady-state ratio of *CYH2* pre-mRNA/mRNA when Upf2 mutants were present with either wild-type Upf1 or Upf1 mutant 27 (defective in Edc3-Upf1 and Upf2-Upf1 interaction). Four different classes of Upf2 mutants were utilized [8]: 1) Mutants which still interact with Upf1 by yeast two-hybrid assay and show wild-type levels of NMD (Upf2 M13). 2) A mutant which showed significant reduction in the Upf2-Upf1 interaction but still showed wild-type levels of NMD (Upf2 M6-NE). 3) A mutant which interacts at wild-type levels with Upf1 but shows a defect in NMD (Upf2 M6-G). 4) Mutants which both impair the Upf2-Upf1 interaction and lead to defects in NMD (Upf2 M5).

Our results suggest that the Edc3-Upf1 interaction does not have an inhibitory role on NMD, as Upf1 mutant 27 did not suppress the NMD defects caused by Upf2 mutants with inefficient NMD (Figure 5C). Rather, Upf1 mutant 27 lead to impairment of NMD in the presence of both wild-type Upf2 and Upf2 mutants that were still able to undergo moderate to normal levels of NMD. In conjunction with Upf1 mutant 27, class 1 Upf2 M13 and class 2 Upf2 M6-NE showed impaired NMD compared to wild-type Upf1 levels. Class 3 Upf2 M6-G showed higher levels of NMD in the presence of wild-type Upf1 in our hands compared to that which was previously published [8], and this was impaired in the presence of Upf1 mutant 27. Lastly, class 4 Upf2 M5, which showed impaired NMD with wild-type Upf1, likewise showed impaired NMD with Upf1 mutant 27. There was a slight suppression of the NMD defect in the presence of Upf1 mutant 27, but not a significant reduction. Thus, it appears that Upf1 mutant 27 leads to further defects in NMD and does not sufficiently suppress defects caused by Upf2 mutants. This provides additional evidence against an inhibitory role for the Edc3-Upf1 interaction.

However, it is possible that Upf1 mutant 27 does not suppress the Upf2 mutants because we have insufficiently disrupted the Edc3-Upf1 interaction by the specific mutations we used in Upf1. Therefore, to be sure that the Edc3-Upf1 interaction is eliminated, we constructed an *edc3*Δ *upf2*Δ strain to test if the loss of Edc3 could suppress the NMD defects of the Upf2 mutants. Loss of Edc3 does not alter the NMD phenotypes of the Upf2 mutants (Figure 5D). Therefore, our results suggests that the Edc3-Upf1 interaction does not impair decapping of NMD targets by blocking Dcp2 function, thereby confirming results obtained by analysis of PGK1c142 mRNA in the absence of Upf2 and results obtained using Upf1 mutant 27 in conjunction with the Upf2 mutants.

## Discussion

### Nature of the NMD decapping complex

In yeast, efficient NMD of aberrant mRNA transcripts is dependent upon the decapping enzyme Dcp2/Dcp1, but the mechanism of how the decapping enzyme associates with the mRNA is unknown. Herein, we have identified interactions between the decapping stimulators, Edc3 and Pat1, and Upf1. Interaction of both of these factors occurs on the N-terminal domain of Upf1. Dcp2 interacts with both Edc3, via the Edc3 Lsm domain, and Pat1 via the middle and C-terminal domains of Pat1 [27], [35]. This raises the possibility that Edc3 and/or Pat1 may be bridging an interaction between Upf1 and Dcp2/Dcp1. Our yeast two-hybrid results suggest that Edc3, but not Pat1, mediates the Upf1-Dcp2 interaction. These results argue that Upf1 can interact with and nucleate a decapping complex that includes interactions of Upf1 with components of a larger decapping complex.

An interesting observation was that the Edc3 and Upf2 binding sites on the N-terminal domain of Upf1 overlap. We constructed a series of mutations along the surface of the Upf1 N-terminal domain and assayed for their ability to interact with both Edc3 and Upf2 by yeast two-hybrid. We find that the Edc3-Upf1 interaction is disrupted when residues at or near one of the two predicted Upf2-binding sites are mutated. One of these five mutants, which contains alanine substitutions at ∼80% of the residues within this predicted Upf2-binding site, also disrupted the Upf1-Upf2 interaction. Further, we confirmed that Upf1 interaction with both Edc3 and Upf2 is not mediated by the opposite factor. This argues that Edc3 and Upf2 share overlapping, although not identical, binding sites on the N-terminal domain of Upf1.

There are two potential models for how Edc3 and Upf2 might interact at an overlapping site on Upf1. One possibility is that these interactions occur on the same mRNA substrate but at a different stage of the NMD process. Alternatively, these interactions may instead occur on different mRNA populations. For example, Upf1 might interact with Edc3 to promote the decay of some yeast mRNAs analogous to how mammalian Upf1 interacts with Staufen in an Upf2-independent manner to recruit Upf1 to a second pool of mRNAs for their degradation [36].

### Significance of Upf1 interaction with decapping stimulators

Upon investigating the role of the interaction of Upf1 with the decapping stimulators, Edc3 and Pat1, we found that neither the Upf1-Edc3 nor the Upf1-Pat1 interactions were essential for NMD. Additionally, we found that Edc3 and Pat1 do not have redundant roles with each other in promoting NMD, as NMD of both a reporter transcript and an endogenous NMD substrate was not altered in an *edc3*Δ* pat1*Δ strain. Upon examining the role of additional decapping stimulators in NMD, we also found that Edc1 and Edc2 were dispensable for NMD. These results strongly argue that decapping stimulators are not required for global NMD in yeast, and raise the possibility that Edc3-Upf1 interaction might regulate a different pool of mRNAs.

One possibility was that the interaction between Edc3 and Upf1 functioned as a negative regulator of NMD. In this model, the Upf1-Edc3 interaction is required to prevent the stimulation of Dcp2 decapping activity on NMD substrates. If this interaction does serve an inhibitory role, some signal must be present to trigger release of Edc3, and subsequently Dcp2, in a temporally dependent manner. The overlapping binding sites of Edc3 and Upf2 on Upf1 suggest that Upf2 may provide the ‘switch’ to trigger release of Edc3 and degradation of the NMD substrate. Upon testing this switch model, we find that the Upf1-Edc3 interaction does not confer negative regulation. Here, disruption of the Upf1-Edc3 interaction did not suppress NMD defects caused by loss of the Upf1-Upf2 interaction. This argues that Edc3 does not negatively regulate NMD, as simple release of Edc3 from Upf1 is not capable of triggering NMD when it is impaired by disrupted Upf1-Upf2 association.

These results raise the question as to the significance of the Upf1 interaction with both Edc3 and Pat1. Additionally, if these interactions are dispensable for NMD, why would loss of Upf2 or Upf3 lead to their accumulation with Upf1 and Dcp2 in cytoplasmic P-bodies [17]? We envision a couple of possible roles for the Upf1-Edc3 and/or Upf1-Pat1 interactions. 1) Edc3 and/or Pat1 might associate with Upf1 to regulate a subset of NMD transcripts. 2) Edc3 and/or Pat1 may be essential for promoting NMD when environmental or growth conditions are altered. It is possible that under normal conditions, Dcp2 is efficiently recruited to an NMD substrate and does not require further assistance from its associating factors. But, under different conditions, a cell may rely upon a broader decapping complex including Edc3 and Pat1, to dramatically increase the levels of NMD transcripts targeted for decapping and subsequent degradation.

## Materials and Methods

### Yeast strains

The yeast strains used in this study, and their genotypes, are found in Table 1. Strains yRP2834, yRP2835, and yRP2836 were constructed by homologous recombination in strain yRP2093. The deletion cassettes were PCR amplified from genomic DNA prepared from the Invitrogen/Resgen Collection strains, yRP2141 (*edc3*Δ), yRP2078 (*upf2*Δ), and yRP2067 (*pat1*Δ) using oligos complementary to the 5′ and 3′ untranslated regions for each open reading frame. Each PCR product was then transformed into yRP2093 and selected for transformants on media containing Geneticin. Yeast strain yRP2837 was constructed by standard laboratory procedure by crossing yRP2078 (*upf2*Δ) with yRP2141 (*edc3*Δ) and verifying the presence of the double deletion by PCR. All yeast strains were transformed using standard laboratory techniques.

**Table 1:** Yeast strains used in this study.

Strains	Properties	References
yRP2093	MATα trp1-901 leu2-3,112 ura3-52 his3-200 gal4Δ gal80Δ LYS2::GAL1-HIS3 GAL2::ADE2 met2::GAL7-lacZ	[37]; provided by Stanley Fields, Yeast Resource Center
yRP2094	MATa trp1-901 leu2-3,112 ura3-52 his3-200 gal4Δ gal80Δ LYS2::GAL1-HIS3 GAL2::ADE2 met2::GAL7-lacZ	[37]; provided by Stanley Fields, Yeast Resource Center
yRP2366	MATa trp1-901 leu2-3,112 ura3-52 his3-200 gal4Δ gal80Δ LYS2::GAL1-HIS3 GAL2::ADE2 met2::GAL7-lacZ plasmid AD-Dpc2 (102–300)	[35]
yRP2365	MATa trp1-901 leu2-3,112 ura3-52 his3-200 gal4Δ gal80Δ LYS2::GAL1-HIS3 GAL2::ADE2 met2::GAL7-lacZ plasmid AD-Dpc2 (1–300)	[35]
yRP2368	MATa trp1-901 leu2-3,112 ura3-52 his3-200 gal4Δ gal80Δ LYS2::GAL1-HIS3 GAL2::ADE2 met2::GAL7-lacZ plasmid AD-Edc3 full-length	[35]
yRP2834	MATα trp1-901 leu2-3,112 ura3-52 his3-200 gal4Δ gal80Δ LYS2::GAL1-HIS3 GAL2::ADE2 met2::GAL7-lacZ edc3Δ::NEO	This Study
yRP2835	MATα trp1-901 leu2-3,112 ura3-52 his3-200 gal4Δ gal80Δ LYS2::GAL1-HIS3 GAL2::ADE2 met2::GAL7-lacZ upf2Δ::NEO	This Study
yRP2836	MATα trp1-901 leu2-3,112 ura3-52 his3-200 gal4Δ gal80Δ LYS2::GAL1-HIS3 GAL2::ADE2 met2::GAL7-lacZ pat1Δ::NEO	This Study
yRP2077	MATa his3Δ1 leu2Δ0 met15Δ0 ura3Δ0 upf1Δ::NEO	Invitrogen/Resgen Collection
yRP2065	MATa his3Δ1 leu2Δ0 met15Δ0 ura3Δ0	Invitrogen/Resgen Collection
yRP2067	MATα his3Δ1 leu2Δ0 lys2Δ0 ura3Δ0 pat1Δ::NEO	Invitrogen/Resgen Collection
yRP840	MATa leu2-3,112 trp1 ura3-52 his4-539 cup1::LEU2/PGK1pG/MFA2pG	[38]
yRP2413	MATa leu2-3,112 trp1 ura3-52 his4-539 cup1::LEU2/PGK1pG/MFA2pG pat1Δ::LEU2	Muhlrad, Denise unpublished
yRP1752	MATa leu2-3,112 trp1 ura3-52 cup1::LEU2/PGK1pG/MFA2pG pat1Δ::LEU2 edc3Δ::NEO	[28]
yRP1745	MATa leu2-3,112 trp1 ura3-52 his4-539 cup1::LEU2/PGK1pG/MFA2pG edc3::NEO	[28]
yRP1503	MATa his3-Δ200 ade2-101 leu2-3,112 lys2-21 trp1 ura3-52 cup1::LEU2/PGK1pG/MFA2pG edc1::HIS3	[24]
yRP1504	MATα leu2-3,112 trp1 ura3-52 his4-539 cup1::LEU2/PGK1pG/MFA2pG edc2::NEO	[24]
yRP1505	MATa leu2-3,112 lys2-21 trp1 ura3-52 cup1::LEU2/PGK1pG/MFA2pG edc1::HIS3 edc2::NEO	Dunckley, Travis unpublished
yRP1754	MATa leu2-3,112 lys2-21 trp1 ura3-52 cup1::LEU2/PGK1pG/MFA2pG edc1::HIS3 edc3::NEO	Doma, Meenakshi unpublished
yRP1755	MATa leu2-3,112 lys2-21 trp1 ura3-52 his4-539 cup1::LEU2/PGK1pG/MFA2pG edc2::NEO edc3::NEO	Doma, Meenakshi unpublished
yRP1756	MATa leu2-3,112 trp1 ura3-52 cup1::LEU2/PGK1pG/MFA2pG edc1::HIS3 edc2::NEO edc3::NEO	Doma, Meenakshi unpublished
yRP2103	MATa his3Δ1 leu2Δ0 met15Δ0 ura3Δ0 upf1Δ::NEO upf2Δ::NEO	[17]
yRP2078	MATa his3Δ1 leu2Δ0 met15Δ0 ura3Δ0 upf2Δ::NEO	Invitrogen/Resgen Collection
yRP2141	MATα his3Δ1 leu2Δ0 lys2Δ0 ura3Δ0 edc3::NEO	Invitrogen/Resgen Collection
yRP2837	MATa his3Δ1 leu2Δ0 lys2Δ0 ura3Δ0 upf2Δ::NEO edc3Δ::NEO	This Study

### Plasmids

The plasmids and oligos used in this study are presented in Table 2 and Table 3, respectively. Plasmids pRP2277, pRP2285, pRP2279, pRP2280, and pRP2284 were constructed by digestion of either pRP1289 (pOBD-II) or pRP1290 (pOAD) with PvuII and NcoI and repaired by homologous recombination using PCR products for Upf1 (N-terminal 1–230), Dcp2 (1–245), Dcp2 (102–245), and Upf2 (C-terminal 157 Amino Acids). All constructed plasmids were verified by sequencing. Plasmids pRP2281 and pRP2282 were constructed from pRP2278 by QuickChange mutagenesis according to the manufacturer's instructions (Stratagene, CA) using oligos oRP1542 with oRP1543 and oRP1544 with oRP1545, respectively. QuickChange mutagenesis was also used to construct the Upf1 mutations in plasmids pRP2286–2290 and pRP2291–2295 from pRP2277 and pRP910, respectively. Oligos oRP1546–1553 were used to construct Upf1 mutant 3, Upf1 mutant 18, Upf1 mutant 23, and Upf1 mutant 24 by one QuickChange reaction each. Upf1 mutant 27 required three QuickChange reactions: (1) Construction of Upf1 mutant 23 using oRP1550 with oRP1551, (2) addition of Upf1 mutant 24 using oRP1552 with oRP1553, (3) and final addition of four alanine substitutions using oRP1554 with oRP1555. All plasmid mutagenesis was verified by sequencing.

**Table 2:** Plasmids used in this study.

Plasmids	Properties	References
pRP1289	pOBD-II (Binding domain empty vector)	[37]; provided by Stanley Fields, Yeast Resource Center
pRP1290	pOAD (Activating domain empty vector)	[37]; provided by Stanley Fields, Yeast Resource Center
pRP2277	BD-Upf1 N-terminus (1–230)	This Study
pRP2278	AD-Dcp2 (102–300)	Recovered from yRP2366, [35]
pRP2279	AD-Dcp2 (1–245)	This Study
pRP2280	AD-Dcp2 (102–245)	This Study
pRP2281	AD-Dcp2 (102–300) Mutant 19: L255A K256A	This Study
pRP2282	AD-Dcp2 (102–300) Mutant 21: E252A L255A K256A	This Study
pRP2283	AD-Edc3 Full-length	Recovered from yRP2368, [35]
pRP2284	AD-Upf2 C-terminus (Ct 157 AA)	This Study
pRP1511	BD-Pat1 C-terminus (Δ10-422)	[32]
pRP2285	AD-Upf1 N-terminus (1–230)	This Study
pRP2286	BD-Upf1 Mutant 3: Q166A E170A L175A W177A	This Study
pRP2287	BD-Upf1 Mutant 18: P111A D112A D114A D117A	This Study
pRP2288	BD-Upf1 Mutant 23: L115A Y123A	This Study
pRP2289	BD-Upf1 Mutant 24: W177A	This Study
pRP2290	BD-Upf1 Mutant 27: L115A Y123A W177A VVVL143AAAA	This Study
pRP249	pRS415 empty vector; cen; leu	[39]
pRP910	Flag-Upf1 wild-type; cen; leu	[17]
pRP1076	gal-PGK1c142; cen; ura	[4]
pRP2291	Flag-Upf1 Mutant 3: Q166A E170A L175A W177A	This Study
pRP2292	Flag-Upf1 Mutant 18: P111A D112A D114A D117A	This Study
pRP2293	Flag-Upf1 Mutant 23: L115A Y123A	This Study
pRP2294	Flag-Upf1 Mutant 24: W177A	This Study
pRP2295	Flag-Upf1 Mutant 27: L115A Y123A W177A VVVL143AAAA	This Study
pRP609	gal-PGK1c103; cen; ura	[3]
pHF713	pRS316-HA-NMD2(X-S)	[8]; Gift from Alan Jacobson lab
pHF889	pRS316-nmd2-M13 (mutations 7958H, K963H, D987G, E1018G, K1024R, Y1027H)	[8]; Gift from Alan Jacobson lab
pHF961	pRS316-nmd2-M6-NE (mutations Y955N, K1010E)	[8]; Gift from Alan Jacobson lab
pHF964	pRS316-nmd2-M6-G (mutations E1070G)	[8]; Gift from Alan Jacobson lab
pHF893	pRS316-nmd2-M5 (mutations S997G, F1061S, I1079T)	[8]; Gift from Alan Jacobson lab

**Table 3:** Oligos used in this study.

Oligo	Sequence	Purpose
oRP1542	AATCCTATGCGGAAGAACAAgcagctTTGTTGTTGGGTATCACTAA	Dcp2 Mutant 19: Mutations L255A K256A
oRP1543	TTAGTGATACCCAACAACAAagctgcTTGTTCTTCCGCATAGGATT	Dcp2 Mutant 19: Mutations L255A K256A
oRP1544	ATCAATTGAAATCCTATGCGgctGAACAAgcagctTTGTTGTTGGGTATCACTAA	Dcp2 Mutant 21: Mutations E252A L255A K256A
oRP1545	TTAGTGATACCCAACAACAAagctgcTTGTTCagcCGCATAGGATTTCAATTGAT	Dcp2 Mutant 21: Mutations E252A L255A K256A
oRP1546	ACTGGGATACTGATCAATGGgctCCATTAATTgctGACAGACAACTTgctTCAgctGTCGCAGAGCAACCAACTGA	Upf1 Mutant 3: Mutations Q166A E170A L175A W177A
oRP1547	TCAGTTGGTTGCTCTGCGACagcTGAagcAAGTTGTCTGTCagcAATTAATGGagcCCATTGATCAGTATCCCAGT	Upf1 Mutant 3: Mutations Q166A E170A L175A W177A
oRP1548	TAACGTAGTTTCTTTACATgcagcaTCTgcaTTAGGGgcaACCGTTTTGGAATGTTATA	Upf1 Mutant 18: Mutations P111A D112A D114A D117A
oRP1549	TATAACATTCCAAAACGGTtgcCCCTAAtgcAGAtgctgcATGTAAAGAAACTACGTTA	Upf1 Mutant 18: Mutations P111A D112A D114A D117A
oRP1550	CTTTACATCCAGATTCTGACgcaGGGGATACCGTTTTGGAATGTgcaAACTGTGGACGTAAGAACGT	Upf1 Mutant 23: Mutations L115A Y123A
oRP1551	ACGTTCTTACGTCCACAGTTtgcACATTCCAAAACGGTATCCCCtgcGTCAGAATCTGGATGTAAAG	Upf1 Mutant 23: Mutations L115A Y123A
oRP1552	AAGACAGACAACTTTTATCAgcaGTCGCAGAGCAACCAACTGA	Upf1 Mutant 24: Mutation W177A
oRP1553	TCAGTTGGTTGCTCTGCGACtgcTGATAAAAGTTGTCTGTCTT	Upf1 Mutant 24: Mutation W177A
oRP1554	TTTCCGCTAAAAGTGAGGCCgcagctgcagcaCTTTGTAGAATACCTTGTGC	Final construction of Upf1 Mutant 27: Mutations VVVL143AAAA
oRP1555	GCACAAGGTATTCTACAAAGtgctgcagctgcGGCCTCACTTTTAGCGGAAA	Final construction of Upf1 Mutant 27: Mutations VVVL143AAAA

### Yeast two-hybrid analysis

Two-hybrid analysis was done in two different manners. The first method relied upon mating of the MATa (yRP2094) and MATα (yRP2093) strains containing specific Activating Domain (AD) or Binding Domain (BD) derivatives, respectively, and selection for diploids on drop-out media. Following this, interaction was assessed using the β-galactosidase plate assay. Assessment of interaction between AD-Dcp2 (102–300) point mutants and BD-Upf1 Nt or AD-Edc3 FL and between BD-Upf1 point mutants and AD-Dcp2 (102–300), AD-Edc3 FL, or AD-Upf2 Ct was done in this manner. The second method relied upon transformation of one strain with both the AD and BD plasmids and selection for these haploids on drop-out media. Following this, the interactions were assessed using the β-galactosidase plate assay. This method was used for assessing interactions in yRP2834 (*edc3*Δ), yRP2835 (*upf2*Δ), and yRP2836 (*pat1*Δ).

### RNA Analyses

Half-life experiments were preformed as previously described [4]. Briefly, cells were grown in selective media containing 2% galactose until OD600 0.3–0.4. Due to the slow growth phenotype of yRP1752, this strain along with the wild-type yRP840 was grown in selective media containing 2% galactose and 1% sucrose until OD600 0.3–0.4. Cells were then harvested and re-suspended in selective media containing 4% glucose to repress transcription of the galactose-inducible reporter transcripts. 2 ml aliquots were then taken at 12 time points over the course of one hour and frozen quickly in liquid nitrogen.

For assessment of *CYH2* steady-state pre-mRNA and mRNA levels, cells were grown in selective media containing 2% glucose until OD600 0.3–0.4. Cells were then harvested and quickly frozen in liquid nitrogen.

For assessment of RNA half-lives and *CYH2* steady-state levels, RNA was extracted by a hot phenol method. RNAs were analyzed by running 10 µg of each sample on a 1.5% formaldehyde agarose gel. PGK1c142 northern analysis was done using the radiolabeled oligo, oRP121. PGK1c103 northern analysis was done using a radiolabeled oligo, oRP252, which is specific for a nucleotide sequence ‘tag’ inserted in frame within PGK1, preventing detection of PGK1pG found within the strains used [3]. *CYH2* pre-mRNA and mRNA transcripts were detected using the radiolabeled oligo, oRP1300. Radiolabeled signal was detected and quantified using a Typhoon phosphoimager (Molecular Dynamics). Determination of half-lives and steady-state levels was done by normalization of each lane to the stable 7S RNA using radiolabeled oligo, oRP100.
